# Supplementary material for: Pharmacological perturbation of the phase-separating protein SMNDC1
Source: Nat Commun. 2023 Aug 16;14:4504. doi: 10.1038/s41467-023-40124-0 (PMC10432564; doi:10.1038/s41467-023-40124-0)
Supplement: Supplementary file 1 — Supplementary Information [file 41467_2023_40124_MOESM1_ESM.pdf]

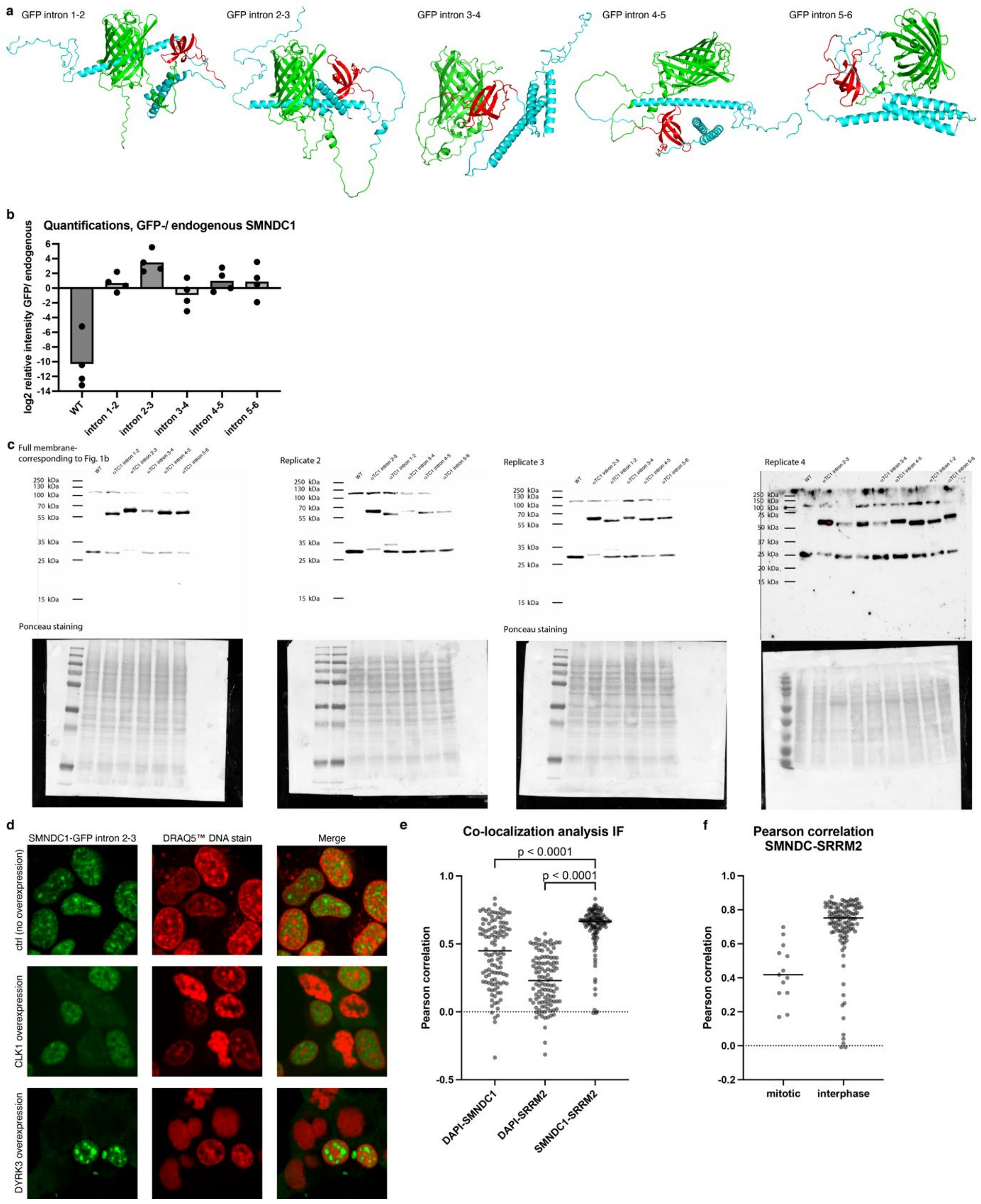

Supplementary Fig. 1 | SMNDC1 co-localizes with nuclear speckle markers. a, AlphaFold structure predictions of SMNDC1 with GFP (marked in green) integrated in different introns. **b**, Quantification of SMNDC1-GFP band divided by endogenous SMNDC1 band in different clonal intron-tag cell lines (αTC1), on a log2 scale. Data shown as mean, n=4. **c**, Immunoblots showing expression of WT SMNDC1 and SMNDC1-GFP fusion proteins in clonal cell lines

with GFP-tag in different introns, 4 replicates. Ponceau staining to show comparable loading. **d**, Live imaging of SMNDC1-GFP intron 2-3 cell line with DRAQ5™ nuclear staining, control (no overexpression), overexpressing CLK1 or DYRK3. **e**, Co-localization analyses of IF images of αTC1 WT with SMNDC1-antibody, SC35-antibody, and DAPI nuclear staining. Pearson correlation between different channels of maximum intensity projections of z-stack images of individual cells (n=114). Data shown as scatter plot + median line, analyzed by two-tailed, unpaired t-test. **f**, Comparison of Pearson correlation values in co-localization analyses of mitotic (n=114) and interphase (n=13) cells, live imaging corresponding to Fig. 1g+h. Data shown as scatter plot + median line.

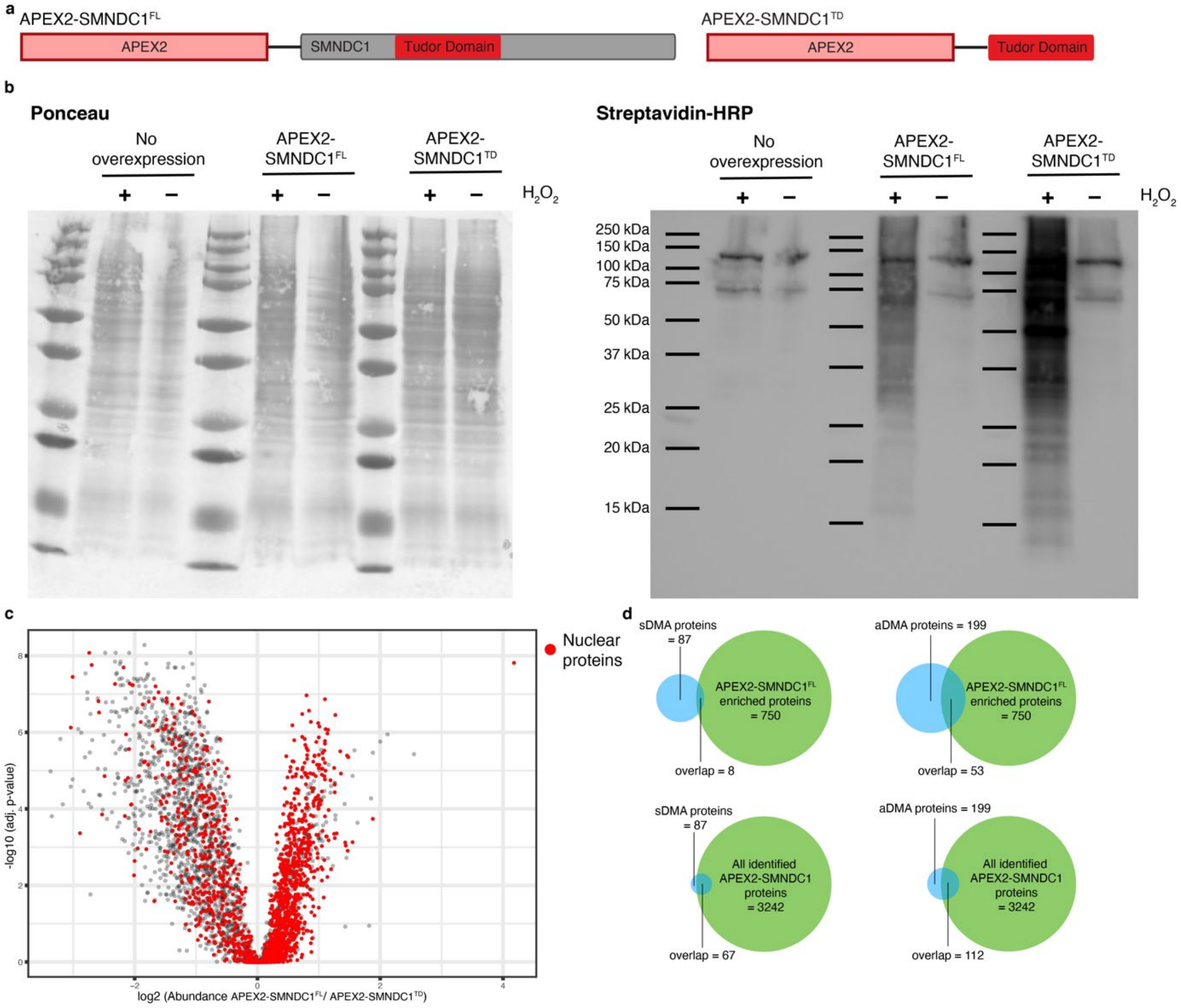

Supplementary Fig. 2 | Characterization of SMNDC1's interactome by proximity labeling. **a**, Depiction of APEX2-fusion constructs APEX2-SMNDC1^{FL} and APEX2-SMNDC1^{TD}. **b**, Ponceau S staining of all proteins and western blot with Streptavidin-HRP showing all biotinylated proteins. **c**, Volcano plot showing log2 abundance against -log10 adjusted p-value of APEX2-SMNDC1^{FL} versus APEX2-SMNDC1^{TD} biotinylated and enriched proteins. Nuclear proteins highlighted in red. **d**, Venn diagrams showing the overlap of all proteins with a known sDMA modification or aDMA modification (light blue), and APEX2-SMNDC1^{FL} enriched proteins or all proteins identified with SMNDC1 proximity labeling (green).

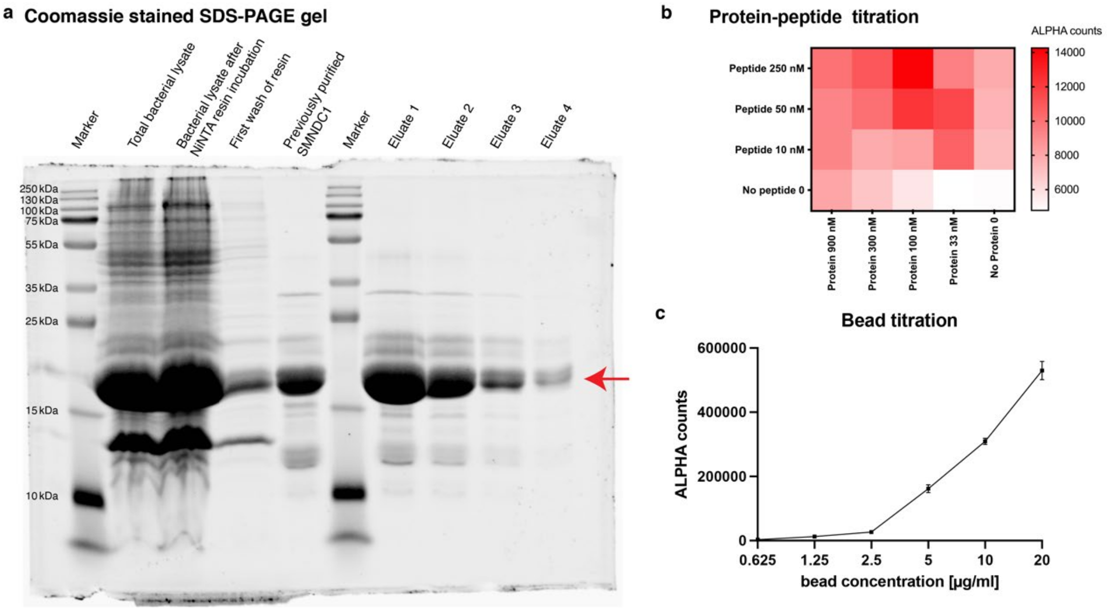

Supplementary Fig. 3 | Establishment of AlphaScreen. a, Coomassie staining of all proteins in different samples along the protein purification process. SMNDC1-Tudor domain marked by red arrow. **b**, Cross-titration of different protein and peptide concentrations in AlphaScreen. **c**, Titration of AlphaScreen acceptor and donor beads with 50 nM peptide, 75 nM protein. Data shown as mean + standard deviation, n=3.

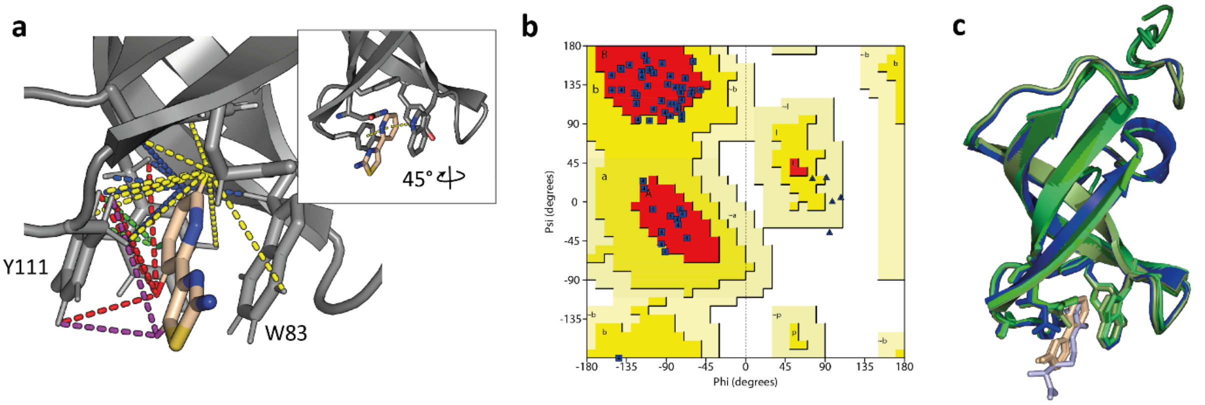

Supplementary Fig. 4 | SMNDC1/inhibitor complex. a, Inter-molecular NOEs (dashed line) used in the restraint-driven docking simulation. NOEs derived from different protons of the inhibitor are colored individually. Phe or Tyr protons HE and HD were not specifically assigned, and a dashed line is only shown for the proton with the closest distance to the NOE contact. The inlet shows the relative orientation of the structure compared to Figure 6. **b**, Ramachandran plot of the four lowest-energy structures, 88.9% and 11.1% of the backbone torsion angles are found in the favored regions and additional allowed regions, respectively. **c**, Overlay of SMNDC1/ compound **13** complex (four lowest-energy structures, shades of green; compound **13**, light brown) and SMNDC1/sDMA (blue/light blue, PDB: 4A4H) shows high structural similarity (backbone rmsd 0.295-0.375 Å).

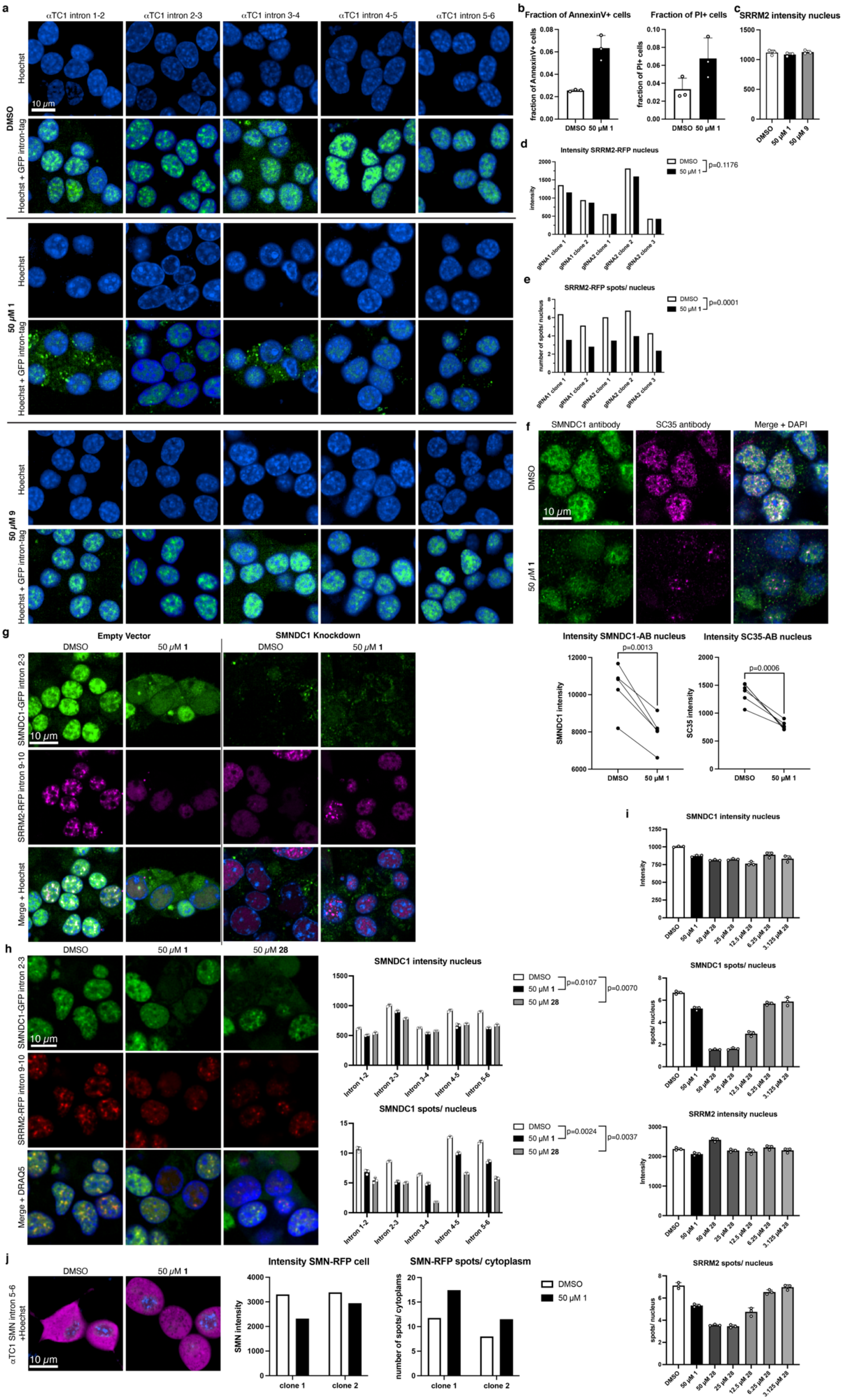
a
αTC1 intron 1-2
αTC1 intron 2-3
αTC1 intron 3-4
αTC1 intron 4-5
αTC1 intron 5-6
DMSO
50 μM 1
50 μM 9
Hoechst
Hoechst + GFP intron-tag
10 μm
b
Fraction of AnnexinV+ cells
Fraction of PI+ cells
DMSO
50 μM 1
c
SRRM2 intensity nucleus
d
Intensity SRRM2-RFP nucleus
p=0.1176
e
SRRM2-RFP spots/ nucleus
p=0.0001
f
SMNDC1 antibody
SC35 antibody
Merge + DAPI
Intensity SMNDC1-AB nucleus
Intensity SC35-AB nucleus
p=0.0013
p=0.0006
g
Empty Vector
SMNDC1 Knockdown
SMNDC1-GFP intron 2-3
SRRM2-RFP intron 9-10
Merge + Hoechst
h
50 μM 28
SMNDC1 intensity nucleus
SMNDC1 spots/ nucleus
p=0.0107
p=0.0070
p=0.0024
p=0.0037
Merge + DRAQ5
i
SRRM2 intensity nucleus
SRRM2 spots/ nucleus
j
αTC1 SMN intron 5-6 +Hoechst
Intensity SMN-RFP cell
SMN-RFP spots/ cytoplasm
clone 1
clone 2

Supplementary Fig. 5 | Effects of SMNDC1 Tudor domain inhibition on localization. a, Live imaging of SMNDC1-GFP αTC1-clones treated with DMSO, compound **1** or **9**. Nuclei stained with Hoechst, corresponding to images in Fig. 7a. **b**, Quantification of AnnexinV+ and PI+ cells (αTC1), treated with DMSO or compound **1**. Data shown as mean + standard deviation, n=3. **c**, Quantification of nuclear SRRM2-RFP in live imaging data of SRRM2-RFP αTC1 cells treated with DMSO, compound **1** or **9**. Data shown as mean + standard deviation, n=3. **d-e**, Quantification of nuclear SRRM2-RFP and SRRM2-RFP spots/ nucleus in live imaging data of different SRRM2-RFP αTC1-clones treated with DMSO or compound **1.** Data analyzed by two-tailed, ratio-paired t-test. **f**, Upper panels: Immunofluorescence images of cells (αTC1 WT) stained with antibodies against SMNDC1 (green), SC25 (magenta), and nuclear marker DAPI (blue), treated with DMSO or compound **1**. Lower panels: Quantifications of SMNDC1-AB intensity and SC35-AB intensity in the nucleus in immunofluorescence imaging data (αTC1), cells treated with DMSO or compound **1**. Data analyzed by two-tailed, ratio-paired t-test. **g**, Representative images of quantifications shown in Fig. 7f. Live imaging data of double-tagged SMNDC1-GFP (intron 2-3) SRRM2-RFP (intron 9-10) αTC1-clone treated with DMSO or compound **1** and transduced with Empty Vector or SMNDC1 knock-down plasmids. **h**, Live imaging data of double-tagged SMNDC1-GFP (intron 2-3) SRRM2-RFP (intron 9-10) αTC1-clone and multiple SMNDC1-GFP clonal cell lines single-tagged in different introns, treated with DMSO, compound **1,** or compound **28.** Quantification of nuclear SMNDC1-GFP and SMNDC1-GFP spots/ nucleus. Data shown as mean + standard deviation, n=3, analyzed by two-tailed, ratio-paired t-test. **i**, Quantification of live imaging data of double-tagged SMNDC1-GFP (intron 2-3) SRRM2-RFP (intron 9-10) αTC1-clone treated with DMSO, 50 µM compound **1,** or 50/ 25/ 12.5/ 6.25/ 3.125 µM compound **28.** Nuclear SMNDC1-GFP, SMNDC1-GFP spots/ nucleus, nuclear SRRM2-RFP, and SRRM2-RFP spots/ nucleus. Data shown as mean + standard deviation, n=3. **j**, Left panel: Live imaging of cells (SMN-RFP intron 5-6, αTC1) treated with DMSO or compound **1**. Quantification of whole cell SMN-RFP and SMN-RFP spots/ cytoplasm in live imaging data of different SMN-RFP cell lines (αTC1).

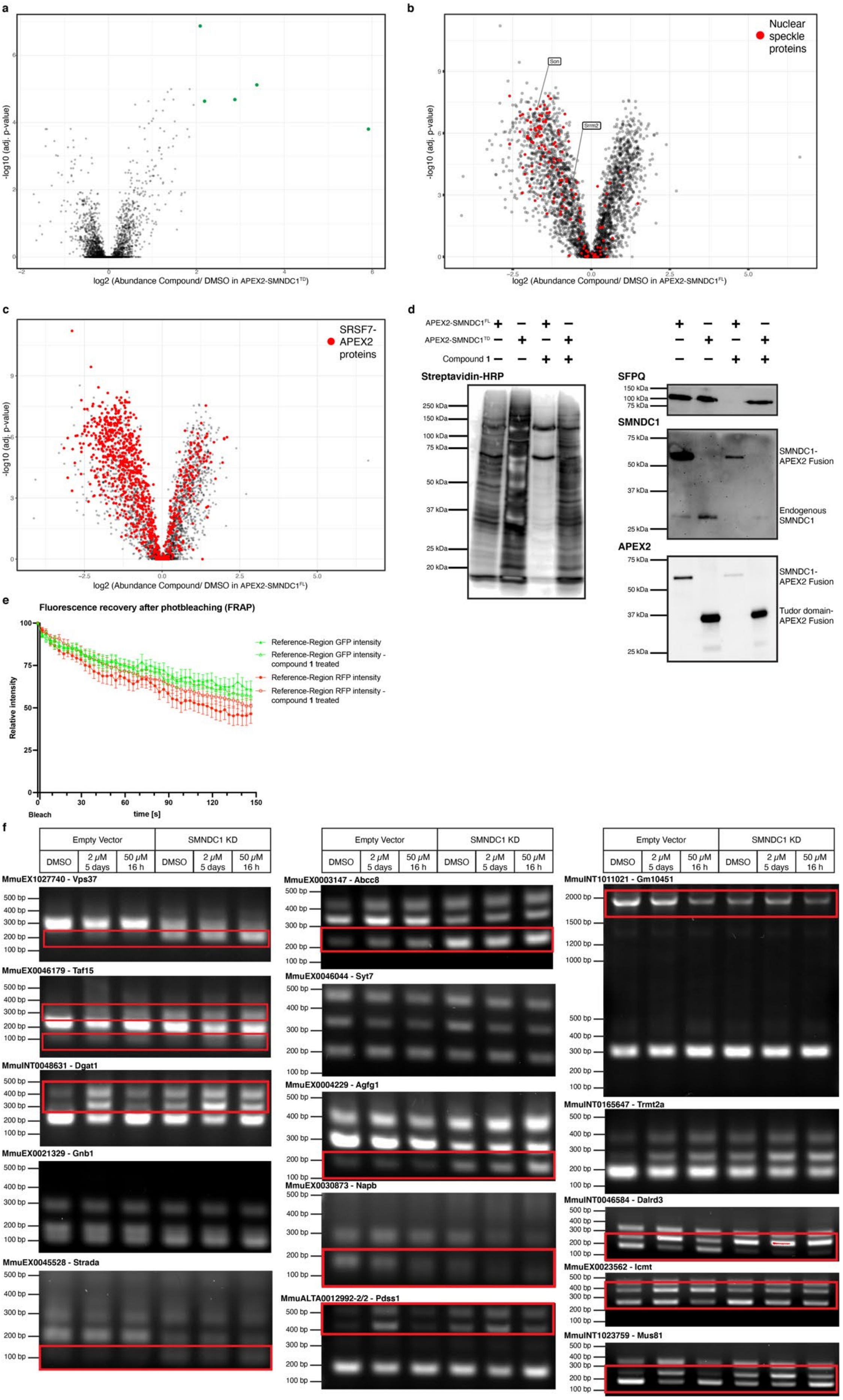
a
-log10 (adj. p-value)
log2 (Abundance Compound/ DMSO in APEX2-SMNDC1TD)
b
Nuclear speckle proteins
Son
Srrm2
log2 (Abundance Compound/ DMSO in APEX2-SMNDC1FL)
c
SRSF7-APEX2 proteins
d
APEX2-SMNDC1FL
APEX2-SMNDC1TD
Compound 1
Streptavidin-HRP
SFPQ
SMNDC1
SMNDC1-APEX2 Fusion
Endogenous SMNDC1
APEX2
Tudor domain-APEX2 Fusion
e
Fluorescence recovery after photbleaching (FRAP)
Reference-Region GFP intensity
Reference-Region GFP intensity - compound 1 treated
Reference-Region RFP intensity
Reference-Region RFP intensity - compound 1 treated
Relative intensity
Bleach
time [s]
f
Empty Vector
SMNDC1 KD
DMSO
2 μM 5 days
50 μM 16 h
MmuEX1027740 - Vps37
MmuEX0046179 - Taf15
MmuINT0048631 - Dgat1
MmuEX0021329 - Gnb1
MmuEX0045528 - Strada
MmuEX0003147 - Abcc8
MmuEX0046044 - Syt7
MmuEX0004229 - Agfg1
MmuEX0030873 - Napb
MmuALTA0012992-2/2 - Pdss1
MmuINT1011021 - Gm10451
MmuINT0165647 - Trmt2a
MmuINT0046584 - Dalrd3
MmuEX0023562 - Icmt
MmuINT1023759 - Mus81

Supplementary Fig. 6 | Effects of SMNDC1 Tudor domain inhibition on interactome and splicing. a, Volcano plot showing log2 protein abundance against -log10 adjusted p-value (one-way ANOVA, Benjamini-Hochberg correction for multiple comparisons) of compound **1** treated cells over DMSO control after APEX2-SMNDC1^{TD} proximity labeling and biotin enrichment. Significantly enriched proteins in green and significantly depleted proteins in red. 0 proteins significantly depleted vs. 5 proteins significantly enriched, adjusted p-value<0.05, |log2FC|≥2. **b**, Volcano plot showing log2 protein abundance against -log10 adjusted p-value (one-way ANOVA, Benjamini-Hochberg correction for multiple comparisons) of compound **1** treated cells over DMSO control after APEX2-SMNDC1^{FL} proximity labeling and biotin enrichment. Nuclear speckle proteins marked in red. **c**, Volcano plot showing log2 protein abundance against -log10 adjusted p-value (one-way ANOVA, Benjamini-Hochberg correction for multiple comparisons) of compound **1** treated cells over DMSO control after APEX2-SMNDC1^{FL} proximity labeling and biotin enrichment. Proteins identified by SRSF7-APEX2 proximity labeling marked in red. **d**, Western blot with Streptavidin-HRP showing all biotinylated proteins, antibodies against SFPQ, APEX2, and SMNDC1. Cells overexpressing APEX2-SMNDC1^{FL} or APEX2-SMNDC1^{TD} were treated with DMSO or compound **1**, and proximity-labeled. Biotinylated proteins were enriched and separated in an SDS-PAGE. Representative images of n=2. **e**, Fluorescence recovery after photobleaching (FRAP) experiment in SMNDC1-GFP intron 2-3, SRRM2-RFP intron 9-10, αTC1-cells, treated with DMSO (filled symbols) or 50 µM compound **1** (empty symbols). Relative intensity of SMNDC1-GFP (green), and SRRM2-RFP (red) only in reference region over time (bleach region: see Fig. 7i). Data plotted as mean with standard error of the mean, n=11 for controls, n=13 for compound 1 treated cells. **f**, DNA-bands on agarose gel after reverse transcription and PCR amplification of RNA to confirm alternative splicing events. RNA was isolated from αTC1 cells transfected with empty vector or SMNDC1 knock-down (KD) plasmid and treated with DMSO, 2 µM compound **1** for 5 days, or 50 µM compound **1** for 16 h. Red boxes show relevant bands for confirmed events.

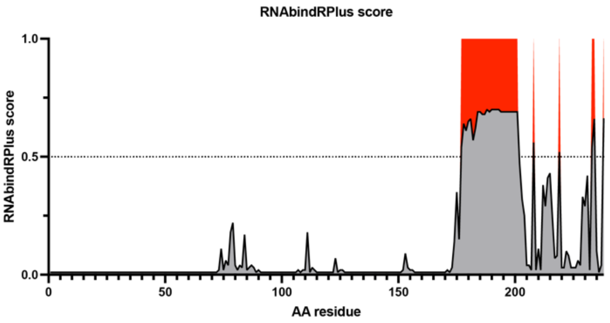

Supplementary Fig. 7 | RNAbindRplus of SMNDC1. RNAbindRplus score of amino acids (AA) over length of SMNDC1 protein. Areas over threshold of 0.5 marked in red.

Supplementary Table 1. Small molecule screening data.

Category	Parameter	Description
Assay	Type of assay	*In vitro* AlphaScreen / luminescence proximity
	Target	Tudor domain of SMNDC1
	Primary measurement	Decrease in luminescence signal reflecting disruption of heterodimeric complex between SMNDC1-Tudor domain and a peptide corresponding to the C-terminus of Small nuclear ribonucleoprotein Sm D3 containing 4 sDMAs (Sequence: AAR*GR*GR*GMGR*GNIFQKRR, R*=sDMA)
	Key reagents	AlphaScreen no-wash assay kit containing Streptavidin Donor beads and nickel chelate (Ni-NTA) AlphaScreen Acceptor beads (PerkinElmer Part Number 6760619).
	Assay protocol	Described in Methods section
	Additional comments	-
Library	Library size	89,355 small molecules
	Library composition	Structural diversity, NIH clinical collection, natural products, approved drugs, known bioactives (e.g., kinase, epigenetic modifiers, ...), natural products, drug-like molecules
	Source	Cayman chemical, Enamine Ltd, LC Labs, MedChem Express, Selleck Chemicals, Sigma Aldrich, Tocris, Toronto Research Chemicals, Chemietek, Merck Millipore, Specs, ChemDiv, Zelinsky
	Additional comments	
Screen	Format	PerkinElmer OptiPlate-384 well plate
	Concentration(s) tested	Typically 10 µM (0.1% DMSO)
	Plate controls	32 positive control wells (Mitoxantrone, quencher), 32 negative control wells (DMSO)
	Reagent/ compound dispensing system	Echo 520 Liquid Handler Multidrop™ Combi Reagent Dispenser
	Detection instrument and software	2104 EnVision Multilabel Plate Reader
	Assay validation/QC	Average Z′-score= 0.821
	Correction factors	-
	Normalization	Raw signal was normalized plate-specifically by correcting row and column-specific mean signals to the mean signal of the entire plate, each after removing the highest and lowest 25% of values. Raw signal was then converted to percent of control signal, but linear regression to plate-specific mean signal of DMSO wells (set to 100 percent of control) and positive control wells (set to 0% of control), after outlier removal using a Grubbs test.
	Additional comments	
Post-HTS analysis	Hit criteria	Percent of control ≤ 50 %
	Hit rate	Primary screen: 511 small molecules (including unspecific quenchers), Secondary screen (with crosslinking peptide): 40 small molecules, Tertiary screen (titration): 14 small molecules.
	Additional assay(s)	Secondary screen with crosslinking peptide, Tertiary screen as 4-point titration, also with related Tudor domain of SMN
	Confirmation of hit purity and structure	Re-ordered and tested selected hits (Fig. 3 f-l)
	Additional comments	

Supplementary Table 2. Overview of all tested small molecules.

	structure	SMNDC1 IC_{50} (µM)	SMN1 IC_{50} (µM)		structure	SMNDC1 IC_{50} (µM)	SMN1 IC_{50} (µM)
**29**	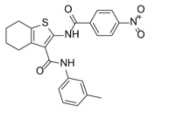	29.16	>50	**41**	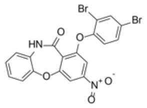	9.90	48.39
**30**	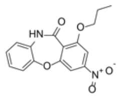	29.27	>50	**42**	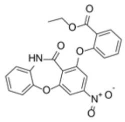	22.57	>50
**31**	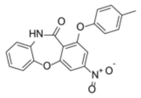	9.51	42.12	**43**	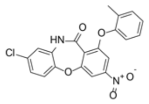	2.41	6.80
**32**	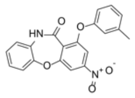	23.91	>50	**44**	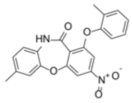	2.02	8.41
**33**	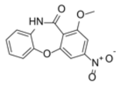	>50	49.95	**45**	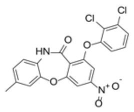	1.14	4.72
**34**	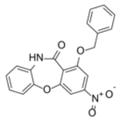	24.91	22.95	**46**	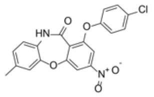	2.38	7.03
**35**	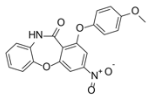	11.27	16.19	**47**	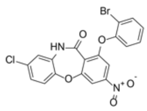	4.43	10.94
**36**	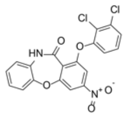	8.61	41.27	**48**	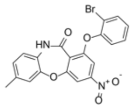	3.43	16.65
**37**	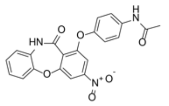	8.56	22.40	**49**	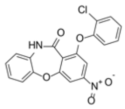	10.61	>50
**38**	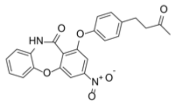	17.84	38.72	**50**	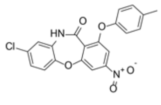	2.17	15.91
**39**	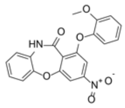	12.53	15.61	**51**	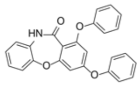	38.37	>50
**40**	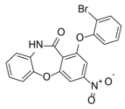	31.15	>50	**52**	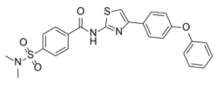	>50	>50

	structure	SMNDC1 IC_{50} (μM)	SMN1 IC_{50} (μM)		structure	SMNDC1 IC_{50} (μM)	SMN1 IC_{50} (μM)
**53**	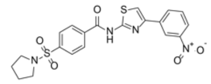	>50	>50	**65**	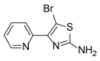	3.05	8.36
**54**	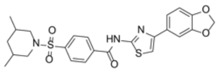	>50	>50	**66**	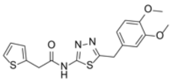	2.33	5.77
**55**	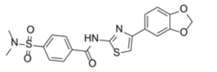	36.72	>50	**67**	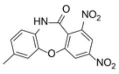	4.13	12.25
**56**	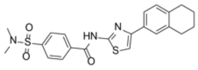	26.52	>50	**68**	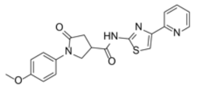	3.01	3.49
**57**	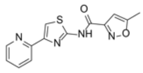	0.47	0.90	**69**	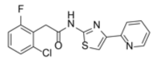	3.06	3.51
**58**	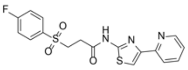	5.15	>50	**70**	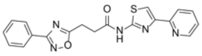	16.83	15.43
**59**	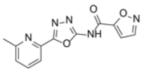	>50	>50	**71**	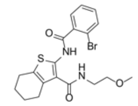	>50	>50
**60**	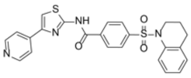	>50	>50	**72**	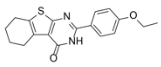	>50	>50
**61**	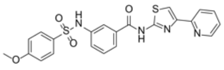	2.82	4.49	**73**	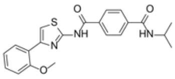	>50	>50
**62**	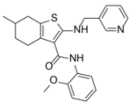	1.53	2.88	**74**	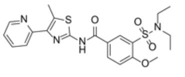	7.35	42.86
**63**	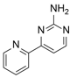	5.89	21.68	**75**	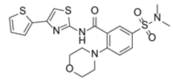	26.05	34.22
**64**	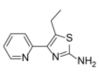	1.18	3.14	**76**	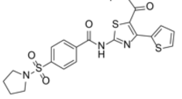	49.92	>50

	structure	SMNDC1 IC_{50} (µM)	SMN1 IC_{50} (µM)
**77**	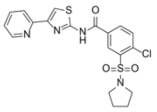	3.47	3.17
**78**	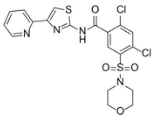	2.47	3.79
**79**	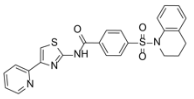	11.23	5.68
**80**	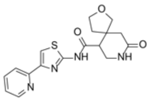	1.84	1.76
**81**	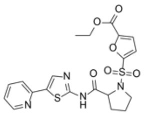	18.51	>50
**82**	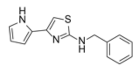	9.60	22.12
**83**	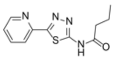	>50	>50
**84**	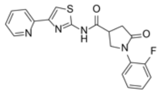	2.43	3.58
**85**	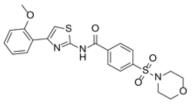	>50	36.93
**86**	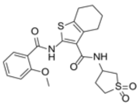	>50	>50

	structure	SMNDC1 IC_{50} (µM)	SMN1 IC_{50} (µM)
**87**	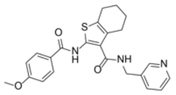	>50	>50
**88**	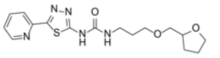	>50	>50
**89**	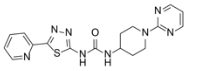	>50	>50
**90**	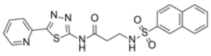	>50	>50
**91**	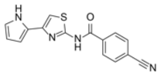	4.82	6.29
**h**	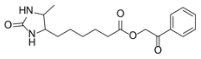	0.19	3.99
**i**	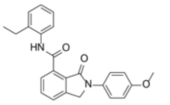	1.44	3.91
**j**	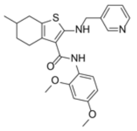	1.81	1.07
**k**	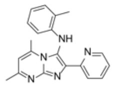	1.09	1.15

Supplementary Table 3. Assigned intermolecular NOEs between SMNDC1 and inhibitor **13** and associated restraint upper limits.

**Protein / inhibitor contacts**		HE-Y111 / H2-**13**	4.6	HE-Y111 / H4-**13**	4.9
HZ2-W83 / H2-**13**	4.7	HB2-W83 / H3-**13**	5.0	HB1-N113 / H4-**13**	5.0
HE-Y90 / H2-**13**	4.4	HB1-Y90 / H3-**13**	5.0	HB2-Y90 / H5-**13**	5.0
HB2-F108 / H2-**13**	5.0	HB2-Y90 / H3-**13**	5.0	HB2-Y111 / H5-**13**	5.0
HE-F108 / H2-**13**	4.7	HB2-F108 / H3-**13**	5.0	HD-Y111 / H5-**13**	4.7
HB1-Y111 / H2-**13**	5.0	HB1-Y111 / H3-**13**	5.0	HE-Y111 / H5-**13**	4.7
HB2-Y111 / H2-**13**	5.0	HD-Y111 / H3-**13**	4.7	HB2-Y111 / H6'-**13**	5.0
HD-Y111 / H2-**13**	4.3	HD-Y111 / H4-**13**	4.6	HE-Y111 / H6'-**13**	4.6

Supplementary Table 4. SMNDC1/ compound **13** molecular-docking results of structure ensemble generated using the HADDOCK webserver[52,53]. Statistics generated over all 200 analyzed structures.

HADDOCK score	-23.1 ± 0.0		
Cluster size	200		
Number of inter-molecular distance restraints	23[a]		
Number of violations (mean)	>0.5 Å: 1[b]	>0.2 Å: 1	>0.1 Å: 2.3
Violations (mean ± standard deviation) (Å)	0.39 ± 0.014		
RMSD from overall lowest-energy structure (Å)	0.6 ± 0.6		
Van der Waals energy	-17.4 ± 0.6		
Electrostatic energy	-15.2 ± 0.5		
Desolvation energy	-7.9 ± 0.2		
Restraint violation energy	37.5 ± 0.14		
Buried Surface Area	312.8 ± 1.4		
Average pairwise backbone RMSD (Å)	0.61 ± 0.79		

[a] Restraints listed in Supplementary Table 3.
[b] The violated distance involves proton **13**-H2 and Y90-HE.
