# Supplementary material for: Pharmacological perturbation of the phase-separating protein SMNDC1
Source: Nat Commun. 2023 Aug 16;14:4504. doi: 10.1038/s41467-023-40124-0 (PMC10432564; doi:10.1038/s41467-023-40124-0)
Supplement: Supplementary file 3 — Reporting Summary [file 41467_2023_40124_MOESM3_ESM.pdf]

nature portfolio

Corresponding author(s): Stefan Kubicek

Last updated by author(s): Jul 6, 2023

Reporting Summary

Nature Portfolio wishes to improve the reproducibility of the work that we publish. This form provides structure for consistency and transparency in reporting. For further information on Nature Portfolio policies, see our Editorial Policies and the Editorial Policy Checklist.

Statistics

For all statistical analyses, confirm that the following items are present in the figure legend, table legend, main text, or Methods section.

n/a	Confirmed	
☐	☒	The exact sample size (*n*) for each experimental group/condition, given as a discrete number and unit of measurement
☐	☒	A statement on whether measurements were taken from distinct samples or whether the same sample was measured repeatedly
☐	☒	The statistical test(s) used AND whether they are one- or two-sided<br>*Only common tests should be described solely by name; describe more complex techniques in the Methods section.*
☒	☐	A description of all covariates tested
☐	☒	A description of any assumptions or corrections, such as tests of normality and adjustment for multiple comparisons
☐	☒	A full description of the statistical parameters including central tendency (e.g. means) or other basic estimates (e.g. regression coefficient) AND variation (e.g. standard deviation) or associated estimates of uncertainty (e.g. confidence intervals)
☐	☒	For null hypothesis testing, the test statistic (e.g. *F*, *t*, *r*) with confidence intervals, effect sizes, degrees of freedom and *P* value noted<br>*Give P values as exact values whenever suitable.*
☒	☐	For Bayesian analysis, information on the choice of priors and Markov chain Monte Carlo settings
☒	☐	For hierarchical and complex designs, identification of the appropriate level for tests and full reporting of outcomes
☐	☒	Estimates of effect sizes (e.g. Cohen's *d*, Pearson's *r*), indicating how they were calculated

Our web collection on statistics for biologists contains articles on many of the points above.

Software and code

Policy information about availability of computer code

Data collection

- Western Blot: Bio-Rad ChemiDoc™ MP with Image LabTM Touch Software Version 2.3.0.07
- Imaging: PerkinElmer Opera Phenix automated microscope high-content image acquisition and analysis software Harmony® 4.9 developed by PerkinElmer
- ALPHAscreen: 2104 EnVision Multilabel Plate Reader
- Mass spectrometry: Mass spectrometry analysis was performed on an Orbitrap Fusion Lumos Tribrid mass spectrometer (ThermoFisher Scientific, San Jose, CA) coupled to a Dionex Ultimate 3000 RSLCnano system (ThermoFisher Scientific, San Jose, CA) via a Nanospray Flex Ion Source (ThermoFisher Scientific, San Jose, CA) interface. Xcalibur version 4.3.73.11 and Tune 3.4.3072.18 were used to operate the instrument.
-NMR: Bruker Avance III spectrometer
-Sequencing: Expression profiling libraries were sequenced on NovaSeq 6000 instrument (Illumina, San Diego, CA, USA)

Data analysis

-AlphaFold: AlphaFold predictions were run via ColabFold (v1.2.0) with the AlphaFold2 algorithm
- Imaging: high-content image acquisition and analysis software Harmony® 4.9 developed by PerkinElmer
-Colocalization analysis: Images were preprocessed in Python version 3.7.9. Z-stacks in czi format were loaded with czifile library, version 2019.7.2, and reduced using maximum intensity Z projection. Segmentation of nuclei was carried out with Cellpose76 (version 0.6.1) based on the DAPI channel. Additional segmentation masks (mitotic nuclei only) were created manually. Preprocessed images and segmentation masks were saved in PNG format. CellProfiler77 (4.0.7) was used to extract fluorescence intensity measurements for non-mitotic and mitotic nuclei separately. All preprocessing code and the CellProfiler pipeline are available at https://github.com/reinisj/colocalization_analysis.
- Mass spectrometry: acquired raw data files were processed using the Proteome Discoverer v.2.4.1.15 platform, choosing a TMT16plex quantification method.
- NMR: Spectra were processed using Topspin 3.5 (Bruker) and analyzed with Cara 1.9.17 or NMRglue-based Python scripts.

- Docking calculations: Docking calculations were performed using the HADDOCK webserver. Structure and topology files for 13 were generated by prodrg2.
- RNA sequencing and transcriptome analysis: RNA sequencing libraries were prepared from low-input samples using the Smart-seq2 protocol87. The subsequent library preparation from the amplified cDNA was performed using the Nextera XT DNA library prep kit (Illumina, San Diego, CA, USA). Library concentrations were quantified with the Qubit 2.0 Fluorometric Quantitation system (Life Technologies, Carlsbad, CA, USA) and the size distribution was assessed using the Experion Automated Electrophoresis System (Bio-Rad, Hercules, CA, USA). For sequencing, samples were diluted and pooled into NGS libraries in equimolar amounts. Expression profiling libraries were sequenced on NovaSeq 6000 instrument (Illumina, San Diego, CA, USA) with a 100-base-pair, paired-end setup. Raw data acquisition and base calling was performed on-instrument. Subsequent raw data processing off the instruments involved two custom programs (https://github.com/epigen/picard/) based on Picard tools (2.19.2) (https://broadinstitute.github.io/picard/). In a first step, base calls were converted into lane-specific, multiplexed, unaligned BAM files suitable for long-term archival (IlluminaBasecallsToMultiplexSam, 2.19.2-CeMM). In a second step, archive BAM files were demultiplexed into sample-specific, unaligned BAM files (IlluminaSamDemux, 2.19.2-CeMM). NGS reads were mapped to the Genome Reference Consortium GRCm38 assembly via "Spliced Transcripts Alignment to a Reference" (STAR)88 utilising the "basic" Ensembl transcript annotation from version e100 (April 2020) as reference transcriptome. The mm10 assembly of the UCSC Genome Browser was used for downstream data processing, and the Ensembl transcript annotations were adjusted to UCSC Genome Browser sequence region names. STAR was run with options recommended by the ENCODE project. NGS read alignments overlapping Ensembl transcript features were counted with the Bioconductor (3.11) GenomicAlignments (1.24.0) package via the summarizeOverlaps function in Union mode, ignoring secondary alignments and alignments not passing vendor quality filtering. Since the Smart-seq2 protocol is not strand specific, all alignments irrespective of the gene or transcript orientation were counted. Transcript-level counts were aggregated to gene-level counts and the Bioconductor DESeq289 (1.28.1) package was used to test for differential expression based on a model using the negative binomial distribution.
-Splicing analysis: Alternative splicing events were characterised and quantified using VAST-TOOLS54 (2.5.1) in conjunction with the Mus musculus database (vastdb.mm2.23.06.20), based on the Genome Reference Consortium assembly GRCm38.p5 and Ensembl transcript annotation 88 (March 2017). Briefly, NGS reads were aligned for each read group independently, read groups were merged into samples and samples were combined into a summary table. The differential splicing events were called via the VAST-TOOLS "compare" algorithm (min_dPSI > 15, min_range > 5) and further filtered for genes showing statistical significance (adjusted P-value <= 0.1) and a sizable effect (absolute log2-fold change >= 1.0) in the differential expression analysis.

For manuscripts utilizing custom algorithms or software that are central to the research but not yet described in published literature, software must be made available to editors and reviewers. We strongly encourage code deposition in a community repository (e.g. GitHub). See the Nature Portfolio guidelines for submitting code & software for further information.

Data

Policy information about availability of data

All manuscripts must include a data availability statement. This statement should provide the following information, where applicable:

- Accession codes, unique identifiers, or web links for publicly available datasets
- A description of any restrictions on data availability
- For clinical datasets or third party data, please ensure that the statement adheres to our policy

Source data are provided with this paper.
The mass spectrometry proteomics data have been deposited to the ProteomeXchange Consortium via the PRIDE partner repository with the dataset identifier PXD037092 and 10.6019/PXD037092 [https://proteomecentral.proteomexchange.org/cgi/GetDataset?ID=PXD037092].
NMR structures have been deposited to PDB with the identifier 8POI [https://www.rcsb.org/structure/8POI] and to BMRB with the identifier 34831 [https://bmrb.io/data_library/summary/?bmrbId=34831].
RNA-seq data have been deposited to GEO with the identifier GSE231600 [https://www.ncbi.nlm.nih.gov/geo/query/acc.cgi?acc=GSE231600].

Research involving human participants, their data, or biological material

Policy information about studies with human participants or human data. See also policy information about sex, gender (identity/presentation), and sexual orientation and race, ethnicity and racism.

Reporting on sex and gender	n/a
Reporting on race, ethnicity, or other socially relevant groupings	n/a
Population characteristics	n/a
Recruitment	n/a
Ethics oversight	n/a

Note that full information on the approval of the study protocol must also be provided in the manuscript.

Field-specific reporting

Please select the one below that is the best fit for your research. If you are not sure, read the appropriate sections before making your selection.

☒ Life sciences ☐ Behavioural & social sciences ☐ Ecological, evolutionary & environmental sciences

Life sciences study design

All studies must disclose on these points even when the disclosure is negative.

Sample size	No sample-size calculations were performed. Sample size was chosen to balance replication and efficiency in the experiments. The exact sample size of the associated experiments was described in the Method and Figure legends. There was in general good correlation between replicates for the different experiments justifying the chosen sample size.
Data exclusions	No data were excluded.
Replication	Number of replicates are described in the legends of the corresponding figures or in the methods section "Statistics and Reproducibility".
Randomization	For cell culture-based experiments, cells were split from the same batch of cells and randomly divided for each treatment in each replicate. Samples per replicate were processed together. Other experiments requiring randomization were not conducted.
Blinding	The investigators were not blinded to allocation during experiments and outcome assessment, as proper controls were already included during the experimental design.

Reporting for specific materials, systems and methods

We require information from authors about some types of materials, experimental systems and methods used in many studies. Here, indicate whether each material, system or method listed is relevant to your study. If you are not sure if a list item applies to your research, read the appropriate section before selecting a response.

Materials & experimental systems

n/a	Involved in the study	
☐	☒	Antibodies
☐	☒	Eukaryotic cell lines
☒	☐	Palaeontology and archaeology
☒	☐	Animals and other organisms
☒	☐	Clinical data
☒	☐	Dual use research of concern
☒	☐	Plants

Methods

n/a	Involved in the study	
☒	☐	ChIP-seq
☒	☐	Flow cytometry
☒	☐	MRI-based neuroimaging

Antibodies

Antibodies used

Primary:
SMNDC1 (western blots) Novus Biologicals Cat#NBP1-47302; RRID:AB_10010256
SMNDC1 (Immunofluorescence) Thermo Fisher Scientific Cat#PA5-31148; RRID:AB_2548622
SC35 GeneTex Cat#GTX11826; RRID:AB_372954
SFPQ Atlas Antibodies Cat#HPA047513; RRID:AB_2680073
APEX2 Innovagen PA-APX2-100

Secondary:
Goat anti-Rabbit IgG Alexa Fluor 546 Thermo Fisher Scientific Cat#A-11010; RRID:AB_2534077
Goat anti-Mouse IgG Alexa Fluor 488 Thermo Fisher Scientific Cat#A-11001; RRID:AB_2534069

Peroxidase AffiniPure Donkey Anti-Mouse IgG Jackson ImmunoResearch Cat#715-035-151; RRID:AB_2340771
Peroxidase AffiniPure Donkey Anti-Rabbit IgG Jackson ImmunoResearch Cat#711-035-152; RRID:AB_10015282
Goat Anti-Chicken IgY H&L (HRP) Abcam ab97135; RRID:AB_10680105

Validation

SMNDC1 (western blots) Novus Biologicals Cat#NBP1-47302; RRID:AB_10010256: Species: Human, Mouse; Applications: WB, IP; Validation: Independent Antibodies, Western Blot
SMNDC1 (Immunofluorescence) Thermo Fisher Scientific Cat#PA5-31148; RRID:AB_2548622: Species: Human, Mouse; Applications: WB, Immunohistochemistry (Paraffin) (IHC (P)), Immunocytochemistry (ICC/IF); Validation: IHC, WB
SC35 GeneTex Cat#GTX11826; RRID:AB_372954: Species: Human, rat, drosophila, dog, monkey, frog, newt; Applications: WB, ICC/IF, IP, ELISA, EM; Validation: -
SFPQ Atlas Antibodies Cat#HPA047513; RRID:AB_2680073: Species: Human; Applications: WB, ICC/ IF, IHC; Validation: Independent Antibodies, RNAi knockdown, IHC, IF
APEX2 Innovagen PA-APX2-100: Species: - (engineered soybean ascorbate peroxidase APEX2 protein); Applications: -; Validation: -

All the antibodies used in this study were obtained from commercial sources and are registered with a Research Resource Identification (RRID) number (see above), except the APEX2 antibody.

Eukaryotic cell lines

Policy information about cell lines and Sex and Gender in Research

Cell line source(s)	AlphaTC1 ATCC Cat#CRL-2934, RRID:CVCL_B036; Parental HAP1 wild-type cells can be obtained from Horizon discovery; Lenti-X 293T (BOSC-23) RRID:CVCL_4401 cell line can be obtained from TakaraBio Cat#632180.
Authentication	Cell lines were not authenticated.
Mycoplasma contamination	All cell lines were tested negative for mycoplasma contamination on a regular basis (once per month).
Commonly misidentified lines (See ICLAC register)	No commonly misidentified cell line (ICLAC register version 11) was used in this study.
